# TRPC channel lipid specificity and mechanisms of lipid regulation

**DOI:** 10.1016/j.ceca.2009.02.006

**Published:** 2009-06

**Authors:** David J. Beech, Yahya M. Bahnasi, Alexandra M. Dedman, Eman AL-Shawaf

**Affiliations:** a Institute of Membrane & Systems Biology, Faculty of Biological Sciences, and Multidisciplinary Cardiovascular Research Centre, University of Leeds, Leeds, LS2 9JT, UK; b Clinical Physiology Department, Faculty of Medicine, Menoufiya University, Egypt

**Keywords:** Cationic channel, Lipid, Transient receptor potential, Calcium

## Abstract

TRPC channels are a subset of the transient receptor potential (TRP) proteins widely expressed in mammalian cells. They are thought to be primarily involved in determining calcium or sodium entry and have broad-ranging functions that include regulation of cell proliferation, motility and contraction. The channels do not respond to a single stimulator but rather are activated or modulated by a multiplicity of factors, potentially existing as integrators at the plasma membrane. This review considers the sensitivity of TRPCs to lipid factors, with focus on sensitivities to diacylglycerols, lysophospholipids, arachidonic acid and its metabolites, sphingosine-1-phosphate (S1P), cholesterol and derivatives, and other lipid factors such as gangliosides. Promiscuous and selective lipid-sensing are apparent. In many cases the lipids stimulate channel function or increase insertion of channels in the membrane. Both direct and indirect (receptor-dependent) lipid effects are evident. Although information is limited, the lipid profiles are consistent with TRPCs having close working relationships with phospholipase C and A2 enzymes. We need much more information about lipid-sensing by TRPCs if we are to fully appreciate its significance, but the available data suggest that lipid-sensing is a key, but not exclusive, aspect of TRPC biology.

## 1 Introduction

TRPCs are a subgroup of the large family of transient receptor potential (TRP) proteins [1–4]. They are predicted to form ion channels and they usually generate ion channel currents on over-expression in cell lines. Like voltage-gated K^+^ channels, they are thought to form channels by gathering around a central ion pore in a group of four subunits, either all of the one type or a mixture of TRPCs (heteromultimeric). Initial studies of over-expressed TRPCs suggested that TRPC1/4/5 and TRPC3/6/7 multimerize as exclusive subgroups, consistent with the observation that these TRPCs also cluster in amino acid sequence comparisons. However, subsequent studies have suggested more flexibility (e.g. [5]) and the composition of native TRPC-containing channels remains an important unsolved problem. Other subclasses of TRP (e.g. TRPP2) may also be involved [6]. TRPC2 stands more alone, being a pseudogene in humans but in rodents acting, at least in part, as a pheromone transduction channel [7].

TRPC-containing channels are permeable to the cations Ca^2+^, Na^+^ and K^+^. They are not voltage-gated (i.e. do not require a change in membrane potential to open) but they are often voltage-sensitive like other TRP channels [1,2]. They may show constitutive activity [8–10] and may be stimulated or inhibited by a range of different chemical or protein factors [4]. They are emerging as polymodal ion channels (i.e. having a multiplicity of activators, modulators or inhibitors) and may exist as integrative sensors of complex chemical signals [4,11]. They are broadly expressed across mammalian cell types. Although some cells express higher amounts of a specific TRPC, all cells seem to express a significant complement of TRPCs. Therefore, TRPCs probably have generic cell functions. TRPC5, for example, has been suggested to have roles in determination of cell shape and movement [12,13]. Nevertheless, specific functions may exist in a context-dependent manner. Functions in physiology and disease are starting to emerge: for example, roles of TRPC6 in familial focal segmental glomerulosclerosis and murine hypoxic pulmonary vasoconstriction [14,15].

Here we focus on lipids as stimulators of mammalian TRPC function. In addition to reviewing the literature we include original data on the lipid-sensitivity of TRPC5 to increase the breadth of information and data on the underlying mechanism by which lysophospholipids stimulate TRPC5. We do not cover regulation by phosphatidylinositolphosphates (e.g. PIP_2_) because it is the topic of other reviews in the issue; nor do we include lipid regulation of other types of TRP channel [16].

## 2 Diacylglycerols (DAGs)

DAGs are composed of two covalently linked fatty acids and may be formed from various sources, one of which is the hydrolysis of PIP_2_. In early studies searching for stimulators of TRPC channels it was observed that DAGs are stimulators of the TRPC3/6/7 subgroup [17]. TRPC2 is also stimulated [18]. Various DAGs are effective, including 1-stearoyl-2-arachidonyl-sn-glycerol [17,19]. The observation has stood the test of time, with DAGs now being used routinely as stimulator of this subclass of TRPCs. DAG stimulation of TRPC6 is not prevented by protein kinase C inhibitors, suggesting it is independent of protein kinase C [17]. Based on computational analysis of amino acid sequences and mutagenesis studies, an N-terminal section of TRPC3/6/7 has been proposed as a lipid-sensing domain that underlies DAG-induced fusion of TRPC3-containing vesicles [20].

The concentrations of exogenous DAGs required to stimulate the channels are relatively high but effects are suggested to be relevant to endogenous DAGs because there is also stimulation by DAG lipase inhibitors [17]. There is also evidence of synergism with IP_3_, potentially conferring greater sensitivity to DAG [21]. The TRPC3/6/7 channels are receptor-activated (i.e. stimulated by agonists at G-protein-coupled receptors), an effect that is thought to arise at least partly because of G-protein stimulation of phospholipase Cβ, cleaving PIP_2_ to generate DAGs. Stretch-activation of TRPC6 may also occur partly through this mechanism [22].

TRPC1 is not thought to be directly stimulated by DAGs, although it should be noted that this TRPC is difficult to study on its own because trafficking to the plasma membrane is often poor in the absence of other co-expressed factors. It may be stimulated by DAGs when co-expressed with TRPC3 [23]. Various studies suggest, however, that TRPC1 is phosphorylated via protein kinase C (which is activated by DAGs) and that endogenous TRPC1-containing channels are stimulated as a consequence [24,25]. TRPC4 and 5 readily traffic to the plasma membrane but, in contrast to TRPC3/6/7, are not stimulated by DAG [17,26]. There is, nevertheless, a suggestion that TRPC5 forms part of a DAG-stimulated channel containing TRPC3 [27]. Furthermore, there is evidence that desensitization following receptor-activation of TRPC4/5 occurs via protein kinase C-dependent phosphorylation [26,28]. Similarly, protein kinase C inhibits TRPC3 [29].

Therefore, DAGs acutely stimulate or enhance insertion of some TRPCs without need for a protein kinase C as an intermediate. DAGs also inhibit TRPCs by triggering protein kinase C-dependent phosphorylation.

## 3 Lysophospholipids

Lysophospholipids such as lysophosphatidylcholine (LPC) are generated by enzymatic action of phospholipase A2s on phosphatidylcholine (PC) and other related substrates. LPC was first shown to be a stimulator of TRPC5 [30]. We have partial insight into the mechanism of this effect. Chemically, the effect lacks head-group specificity because replacement of choline with inositol (LPI) did not affect activity [30]. However, the length of the carbon side-chain was important, suggesting necessity of solubilisation of the lysophospholipid in the lipid bilayer. Because of this solubilisation property, exogenous LPC has detergent effects on lipid bilayers (hence ‘lyso’, indicating cell lysis). However, stimulation of TRPC5 occurs at lower concentrations and is associated with the distinctive current–voltage relationship (*I*–*V*) of TRPC5, showing it relates to the TRPC5 channel rather than non-specific bilayer disturbance.

LPC is suggested to be a ligand at G-protein-coupled receptors [31] but stimulation of TRPC5 by LPC does not require G-protein signaling [30]. Furthermore, LPC stimulates TRPC5 in excised outside-out membrane patches in the absence of GTP, suggesting it acts relatively directly at the channel. This observation has raised an interesting parallel with the TREK1 K^+^ channel, which is also activated by LPC and shows polymodality [32], like TRPC5 [11]. One of the other activators of TREK1 is membrane stretch and an elegant case has been built around the idea that the conical shape of LPC leads to convex membrane curvature on insertion in the outer leaflet of the lipid bilayer, mimicking membrane stretch and activating TREK1 [32]. Does LPC stimulate TRPC5 through a similar mechanism? We have made several observations that make this unlikely.

**Fig. 1:**
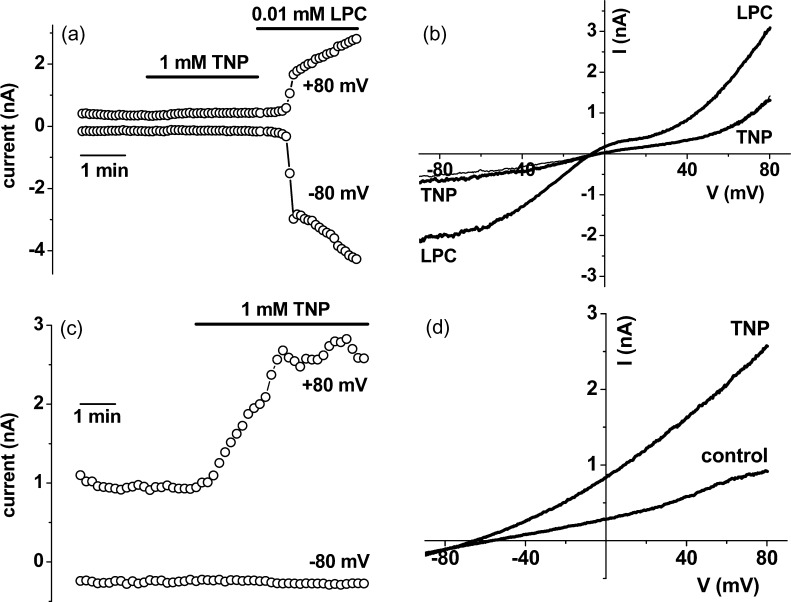
Mechanism of lysophospholipid stimulation of TRPC5. (a and b) Whole-cell currents from HEK 293 cells expressing human TRPC5 with Cs^+^ in the patch pipette to remove K^+^-currents. (a) Time-series for currents sampled at −80 and +80 mV, showing the effects of bath-application of 1 mM 2,4,6-trinitrophenol (TNP) and then 10 μM C18:1 lysophosphatidylcholine (LPC). (b) Current–voltage relationships (*I*–*V*s) showing lack of effect of TNP (the thin trace evident at −80 mV was sampled before TNP application) but effect of LPC with the distinctive TRPC5 shape to the *I*–*V*. (c and d) Whole-cell currents from HEK 293 cells expressing human TREK-1 with K^+^ in the patch pipette. (c) Time-series for currents sampled at −80 and +80 mV, showing bath-application of 1 mM 2,4,6-trinitrophenol (TNP). (d) Current–voltage relationships (*I*–*V*s) showing TNP activation of K^+^-current with the expected *I*–*V* of TREK1. For methods and other additional information see [33].

One observation was with trinitrophenol, an amphipathic substance that preferentially incorporates in the outer leaflet of the lipid bilayer and is said to crenate the plasma membrane (i.e. create a notched appearance). Trinitrophenol failed to stimulate TRPC5 in cells where TRPC5 was subsequently stimulated strongly by LPC (Fig. 1a and b). As expected [32], trinitrophenol activated TREK1 (Fig. 1c and d). Similarly, arachidonic acid is suggested to activate TREK1 through incorporation in the outer leaflet of the bilayer [32] and yet arachidonic does not stimulate TRPC5 [30]. A second observation is with a type of membrane stretch. As expected [32] we observed activation of TREK1 by LPC or convex membrane curvature induced by negative pressure applied to cell-attached patches; however, although expression of TRPC5 conferred greater current in response to negative pressure, the current lacked the hallmark *I*–*V* of TRPC5 [33].

Therefore, chemical (trinitrophenol) and physical (negative pressure) methods that induce convex membrane curvature failed to stimulate TRPC5. These data suggest that TRPC5 does not have intrinsic sensitivity to convex membrane curvature and, therefore, that any convex membrane curvature induced by lysophospholipids does not explain the stimulatory effect of lysophospholipids at TRPC5. Although TRPC5 is stimulated by cell-swelling evoked by hypo-osmotic shock or positive pressure inside cells [34], such stimuli are different from convex membrane curvature and may activate second messenger pathways that couple to TRPC5.

Consistent with the negative data from convex membrane curvature experiments, LPC applied to the inner face of the lipid bilayer also stimulated TRPC5 [30]: that is, the effect of LPC on TRPC5 lacked polarity—acting similarly whether applied to the outside or inside of the membrane. This result is consistent with membrane-spanning elements of TRPC5-containing a lipid interaction site accessible from either side of the membrane, conferring on the open configuration sensitivity to changes in lipid composition of the bilayer.

TRPC6-containing channels are suggested to be stimulated by LPC in endothelial cells [35]. The *I*–*V* of the activated current lacked the distinctive rectification of TRPC channels but responses were reduced when TRPC6 expression was suppressed or prevented, suggesting TRPC6 was involved, but not alone. Biochemical evidence was presented for forward trafficking of TRPC5 in response to LPC-evoked TRPC6-dependent Ca^2+^-entry. In these cells, TRPC5 expression at the plasma membrane was initially low, which might explain why there was no obvious stimulation of TRPC5 in the absence of TRPC6.

LPC stimulation of TRPC channels has importance in endothelial cell migration [35]. It may also have wider importance: human monocytes, for example, show Ca^2+^ entry in response to LPC that is independent of G-protein and phospholipase C signaling and dependent on LPC carbon chain length [36]. Human monocytes express TRPC5 and other TRPCs [27]. A role of TRPC stimulation by LPC has been suggested in erectile dysfunction [37]. Also, store-depletion or agonists acting at G-protein-coupled receptors elevate intracellular LPC and other lysophospholipids [38], raising the possibility that sensitivities of TRPCs to these stimuli reflect sensitivities to lysophospholipids. Lastly, LPC is a major component of oxidized low-density lipoprotein (oxLDL), which may explain Ca^2+^-influx and apoptosis induced by oxLDL in vascular smooth muscle cells [39].

Therefore, lysophospholipids stimulate TRPC channels, apparently relatively directly. The effects are relevant to endogenous concentrations of lysophospholipids and would seem to be important in wide-ranging biological phenomena, both in terms of intracellular and extracellular signaling.

## 4 Arachidonic acid and metabolites

Arachidonic acid is a polyunsaturated fatty acid of lipid bilayers which is generated by phospholipase enzymes and is the precursor for many active metabolites. There are reports that TRPCs are modulated by arachidonic acid and some of its metabolites. Basora et al. [40] reported direct activation of TRPC6 by arachidonic acid and its metabolite 20-HETE. The *I*–*V*s of the activated currents resembled those of TRPC6 only at high 20-HETE concentrations. Intriguingly, no Ca^2+^-entry was evoked by 20-HETE despite the fact that current was observed. Ben-Amor et al. [41] reported block by anti-TRPC1 antibody of Ca^2+^ entry evoked by 5,6-EET in human platelets. Fleming et al. [42] reported surface trafficking of TRPC6 in response to 11,12-EET and pulmonary vasoconstriction evoked by 11,12-EET was less in lungs from TRPC6−/− compared with TRPC6+/− mice [43].

Wu et al. [44] suggested contribution of endogenous TRPC4 to arachidonic acid-evoked Ca^2+^-entry in HEK 293 cells. We have, however, found no effect of arachidonic acid on TRPC5 over-expressed in HEK 293 cells [30], contrasting with effects of the same arachidonic acid on TREK1 and Ca^2+^-activated K^+^ channels (Flemming, Al-Shawaf and Beech, unpublished data). TRPC5 is, nevertheless, stimulated by the arachidonic acid metabolite prostaglandin E2 acting at the EP1 G-protein-coupled receptor [45]. Suppression of TRPC7 expression inhibited apoptosis evoked by prostaglandin E2 in leukaemia cells [46].

In summary, metabolites of arachidonic acid have emerging importance as stimulators of the mammalian TRPC channels. The direct stimulatory effects of arachidonic acid and other polyunsaturated fatty acids evident with *Drosophila* TRP channels [16] have not been described.

## 5 Sphingosine-1-phosphate (S1P)

S1P is generated from sphingosine, which derives from sphingomyelin, a constituent lipid of microdomains in the plasma membrane. TRPC5 is stimulated by intracellular or extracellular S1P [13]. S1P applied to the intracellular surface stimulates TRPC5 in inside-out membrane patches. TRPC5 is, therefore, an intracellular target for S1P but without known physiological importance. van Rossum et al. [47] observed S1P binding to a putative TRPC3-PLCγ1 intermolecular domain that also interacts with phosphatidylinositol phosphates; the functional relevance of this binding is unknown.

Unlike LPC, the extracellular effect of S1P on TRPC5 occurs via G-protein-coupled receptors, illustrating the potential importance of TRPC activation via receptors that have lipids as their ligands. S1P receptors are widely expressed, including in HEK 293 cells often used for TRPC5 over-expression. S1P has no effect on TRPC5 studied in excised outside-out patches without GTP in the pipette. Therefore, unlike LPC, S1P has no direct extracellular effect on TRPC5. The S1P-receptor effect on TRPC5 is functionally important in cell motility and occurs through a G_i/o_ protein pathway [13].

Therefore, S1P is an example of a lipid factor that stimulates TRPC channels via a G-protein-coupled receptor. Suggested intracellular actions of S1P could be biologically important but remain relatively little explored.

## 6 Cholesterol and derivatives

Cholesterol is a constituent sterol lipid of the plasma membrane. Its depletion with methyl-β-cyclodextrin has been shown to suppress store-operated Ca^2+^ signals and ionic current linked to TRPC1 [48–50]. Similarly, cholesterol-loading of cells was found to have a positive effect on signals relating to TRPC3 [51]. TRPC1 has been associated with cholesterol-containing caveolae and lipid rafts [52] and suggested to function as a component of store-operated channels only when linked to STIM1 in lipid rafts [50]. Several studies have linked TRPC1 with caveolins [39,48,52,53]. An elegant study by Huber et al. [54] showed enhancement of TRPC6 by the cholesterol binding protein podocin, dependent on cholesterol binding by podocin. Cholesterol depletion with methyl-β-cyclodextrin inhibited the effect of podocin on TRPC6.

Cholesterol is the precursor for steroid hormones. TRPC2 was recently shown to be activated by sulphated steroids, an effect suggested to have functional importance in odor sensation of rodents [55].

The data suggest dependence of TRPC channels on cholesterol and modulation of TRPC function by localisation to lipid rafts. Effects of cholesterol derivatives are an emerging possibility.

## 7 Other lipid factors

**Fig. 2:**
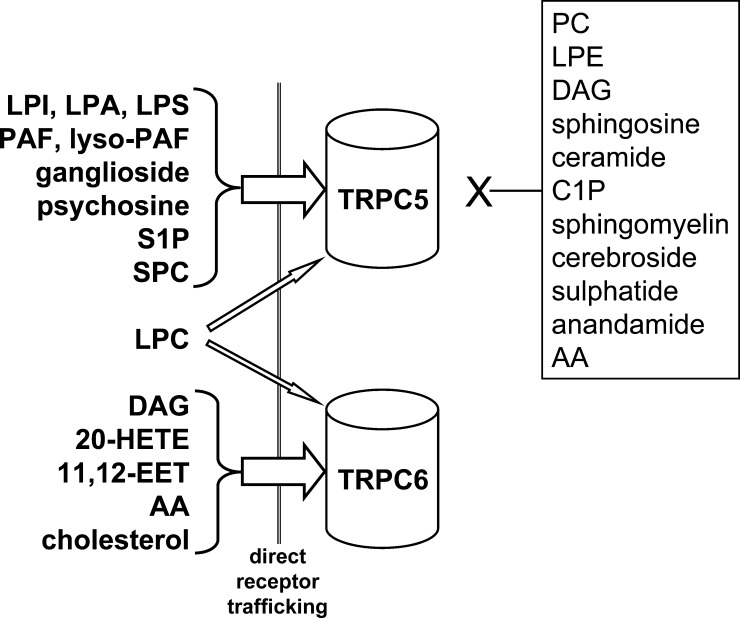
Summary of current knowledge of lipid sensitivities of TRPC5 and TRPC6. Lipids were considered as potential stimulators only. The text should be read for information on whether the lipids act directly or indirectly through G-protein-coupled receptors or membrane trafficking. The box on the right contains names of lipid factors we have found to be ineffective as TRPC5 stimulators at a concentration of 3–10 μM [33] (Bahnasi and Beech, unpublished data). *Abbreviations*: LPC, lysophosphatidylcholine; LPI, lysophosphatidylinositol; LPA, lysophosphatidic acid; LPS, lysophosphatidylserine; LPE, lysophosphatidylethanolamine; PC, phosphatidylcholine; PAF, platelet-activating factor; S1P, sphingosine-1-phosphate; SPC, sphingosylphosphorylcholine; DAG, diacylglycerol; 20-HETE, 20-hydroxyeicosatetraenoic acid; 11,12-EET, 11,12 epoxyeicosatrienoic acid; C1P, ceramine-1-phosphate; AA, arachidonic acid.

Using an intracellular Ca^2+^ assay with HEK 293 cells conditionally over-expressing TRPC5 we have investigated additional lipid factors as potential acute stimulators (Fig. 2). Several lysophospholipids were effective, including the important signaling lipid lysophosphatidic acid (LPA), but not lysophosphatidylethanolamine (LPE) or phosphatidylcholine (PC). Platelet-activating factor (PAF) and lyso-PAF (which is inactive at PAF receptors) were stimulators at 3 μM concentration; both are chemically similar to LPC. Although S1P was a stimulator, sphingosine, sphingomyelin, ceramide and ceramide-1-phosphate (C1P) were not. Sphingosylphosphorylcholine (SPC) was, by contrast, a strong stimulator, where as gangliosides and psychosine were modest stimulators. Cerebrosides, sulphatides and anandamide (an arachidonic acid metabolite) failed to stimulate.

We did not find a stimulatory effect of C1P on TRPC5 but it was recently reported that ceramide kinase and TRPC1 colocalise in cavealae [56], raising the possibility that endogenous TRPC complexes may be sensitive to C1P. Direct tests of this hypothesis are required.

Gangliosides are glycosphingolipids containing sialic acid. Wu et al. [57] have developed an intriguing hypothesis where cross-linking of the GM1 ganglioside with multivalent ligands activates endogenous TRPC5-containing channels via α5β1 integrin. The effect was shown to be important in neuronal growth cone formation.

In a human leukaemia T cell-line, Ca^2+^-entry evoked by Δ^9^-tetrahydrocannabinoid (a lipid-soluble plant-derived cannabinoid) was suppressed when TRPC1 was down-regulated by RNA interference [58]. The effect occurred through cannabinoid G-protein-coupled receptors.

Therefore, there is an emerging breadth to the spectrum of lipids that modulate TRPC channels but also evidence of specificity. There is a great deal more work to be done to determine the full lipid profiles.

## 8 Anaesthetics

Anaesthetics may, in part, modulate ion channel function by disturbing the lipid bilayer. Consistent with this perspective, the LPC-activated TREK1 channel is activated by general anaesthetics [59]. TRPC5 is also sensitive to general anaesthetics but the dominant effect is inhibition of channel function, opposite to the effect of LPC [60]. There is also the peculiar finding that TRPC5 stimulated by LPC is resistant to the anaesthetic propofol, where as TRPC5 stimulated by gadolinium is strongly inhibited. We have interpreted this result as suggesting that propofol does not directly inhibit TRPC5 but compromises a signaling pathway that is necessary for TRPC5 stimulation by the lanthanide [60]. Therefore, there is a complex and poorly understood relationship between anaesthetics and TRPC5. It is not known if other TRPC channels are sensitive to anaesthetics.

## 9 Conclusions

TRPCs have emerged as a class of lipid-sensing cationic channel. Understanding remains elementary but, in some instances, we may start to consider them as lipid ionotropic receptors. In other instances, however, lipids are ineffective or act indirectly via separate receptor proteins. It should be recognized that TRPCs can also be activated by non-lipid factors, including extracellular acid or redox protein [10,61], and can exhibit significant constitutive activity [8–10]. Therefore, although TRPCs are responsive to lipid factors, they may not depend on them.

Fig. 2 summarises knowledge of lipids that stimulate TRPC5 or TRPC6. The figure is limited to stimulatory effects of lipids because there is relatively little information on inhibition. It also focuses on TRPC5 and TRPC6 as examples of two TRPC subtypes about which we have most information. The clearest distinction between the channels is stimulation of TRPC6 but not TRPC5 by DAG but, unfortunately, a comparison is lacking for most lipids. There is promiscuity in the lipid-sensitivity but also selectivity, with a considerable number of lipids failing to stimulate TRPC5. The lipid profiles are consistent with intricate relationships of TRPCs with activities of phospholipase C and A2 enzymes.

Further investigation of TRPC modulation by lipid factors is important because a primary role of TRPCs could be lipid-sensing and major lipid-driven disease states may be explained by over-stimulation or vesicle fusion of TRPCs. We need much more information about the topic in order to make substantial conclusions. In many cases the lipid-sensing profile of the TRPC is limited or we have information only about TRPCs over-expressed in cell-lines rather than endogenous TRPC complexes. In most cases the mechanism of action of the lipids is unknown or superficially understood, and potential for synergy between actions of lipids has been little explored. Experimenters will be familiar with the difficulties of working with lipids because of solubility, stability and detergent problems, let alone complexities of endogenous metabolism once exogenous lipids come into contact with cells. Nevertheless, if we are to understand TRPC channels we need to take on these challenges in the years to come.

## Conflict of interest

None.
